# JDSNMF: Joint Deep Semi-Non-Negative Matrix Factorization for Learning Integrative Representation of Molecular Signals in Alzheimer’s Disease

**DOI:** 10.3390/jpm11080686

**Published:** 2021-07-21

**Authors:** Sehwan Moon, Hyunju Lee

**Affiliations:** School of Electrical Engineering and Computer Science, Gwangju Institute of Science and Technology, Gwangju 61005, Korea; sehwanmoon@gm.gist.ac.kr

**Keywords:** multi-omics, deep learning, Alzheimer’s disease, matrix factorization, feature reduction, feature engineering

## Abstract

High dimensional multi-omics data integration can enhance our understanding of the complex biological interactions in human diseases. However, most studies involving unsupervised integration of multi-omics data focus on linear integration methods. In this study, we propose a joint deep semi-non-negative matrix factorization (JDSNMF) model, which uses a hierarchical non-linear feature extraction approach that can capture shared latent features from the complex multi-omics data. The extracted latent features obtained from JDSNMF enabled a variety of downstream tasks, including prediction of disease and module analysis. The proposed model is applicable not only to sample-matched multiple data (e.g., multi-omics data from one cohort) but also to feature-matched multiple data (e.g., omics data from multiple cohorts), and therefore it can be flexibly applied to various cases. We demonstrate the capabilities of JDSNMF using sample-matched simulated data and feature-matched multi-omics data from Alzheimer’s disease cohorts, evaluating the feature extraction performance in the context of classification. In a test application, we identify AD- and age-related modules from the latent matrices using an explainable artificial intelligence and regression model. These results show that the JDSNMF model is effective in identifying latent features having a complex interplay of potential biological signatures.

## 1. Introduction

With the development of high-throughput technology, large amounts of omics data have been produced. These resources enable us to understand disease- and patient-specific variations. Each omics datapoint contains different forms of information and is complementary to other data. By integrating multi-omics data, we can capture more complex interactions of the biological system.

Motivated by this, many studies using various machine learning methods have been performed to integrate multi-omics data, including MOFA [1] based on factor analysis, MSFA [2] based on factor analysis, RGCCA [3] based on canonical correlation analysis, joint NMF (jNMF) [4] based on non-negative matrix factorization (NMF), integrative NMF [5] based on NMF, intNMF [6] based on NMF, iCluster [7] based on Gaussian latent variable model, and JIVE [8] based on principal component analysis. A common aim of these approaches is to characterize heterogeneity between samples. However, these approaches have two limitations. First, non-linear relationships between features can be missed. Second, these approaches require matching samples of multiple omics data. Analysis can be performed only for samples having complete multi-omics data.

**Figure 1:**
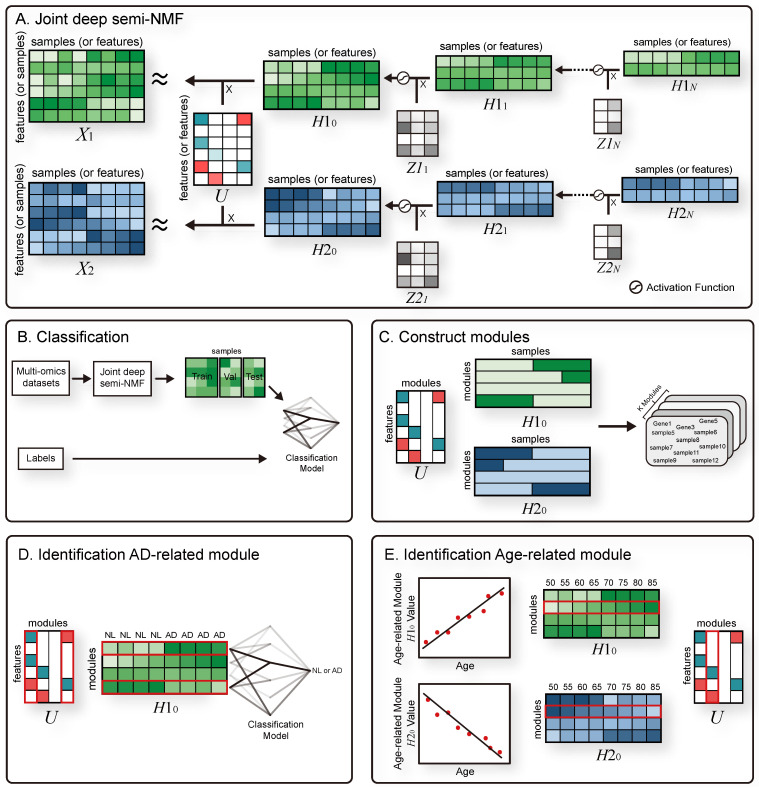
Joint deep-semi NMF (JDSNMF) overview and downstreamanalyses. (**A**)Model overview: JDSNMF decomposes feature-matched (or sample-matched) multiple data. Taking Alzheimer’s disease experiments as an example, JDSNMF decomposes gene expression (GE) (*X*1) and DNA methylation (DM) (*X*2) data into a feature latent matrix (*U*), GE sample latent matrices (*H*1_0_, …, *H*1*_N_*), DM sample latent matrices (*H*2_0_, …, *H*2*_N_*), and junction latent matrices (*Z*1_1_, …, *Z*1*_N_*, *Z*2_1_, …, *Z*2*_N_*). (**B**) JDSNMF is used to integrate multi-omics datasets to latent matrices. These latent matrices, which capture the potential biological signatures that should be used in classification, are fed into the machine learning algorithmto generate a classification model. (**C**) Modules of features and samples were constructed using a feature latent matrix (*U*), a sample latent matrix of GE (*H*1_0_), and a sample latent matrix of DM (*H*2_0_). In *U*, *H*1_0_, and *H*2_0_, cyan, green, and blue elements were larger than positive thresholds, and in *U*, red elements were smaller than a negative threshold. (**D**,**E**) Biologically meaningful modules identified using an explainable artificial intelligence (xAI) and regression model.

To enhance the understanding of complex relationships from multi-omics data, we developed a novel method called the joint deep semi-NMF model (JDSNMF) (Figure 1A). This model is an improvement for multi-omics data over the deep semi-NMF algorithm [9], which is a non-linear feature extraction approach for a single data type in computer vision tasks. JDSNMF is applicable to multiple data with matching features or samples. Cohorts that do not have multi-omics data for a specific disease can be used for integrated analysis. JDSNMF can also be applied to various scenarios such as cross-species or cross-disease comparisons. We demonstrate the capability of JDSNMF and compare its feature extraction performance in the context of classification using simulated data and publicly available Alzheimer’s disease (AD) datasets (Figure 1B). In simulated experiments, we evaluate the feature extraction performance of JDSNMF on multiple data (sample-matched). In real dataset experiments, we evaluate the feature extraction performance of JDSNMF on multi-omics data (feature-matched). In addition we demonstrate the interpretability of JDSNMF on feature-matched AD datasets (Figure 1C,E).

In summary, the main contributions of this work include:

1. It introduces a hierarchical non-linear unsupervised learning model for capturing complex information from multi-omics data.
2. It introduces a flexible integration model for multiple data which does not require matching samples.
3. We compared the feature extraction performance of JDSNMF using sample-matched simulated data and feature-matched AD data.
4. We identified the interpretability of JDSNMF through module analysis using an explainable artificial intelligence (xAI) (Figure 1D) and regression model (Figure 1E).

## 2. Related Work

In this section, a brief review of the most relevant works, i.e., NMF, joint NMF, semi-NMF, and deep semi-NMF, is provided.

### 2.1. Non-Negative Matrix Factorization

NMF [10,11] is a matrix factorization algorithm that contains non-negative constraints at the element of all matrices. It is a widely used linear feature extraction method in many fields. NMF approximately decomposes $X\approx Z^{+}H^{+}$ by minimizing the following objective function.

(1) $$\begin{array}{c} \min_{Z,H}{∥X-ZH∥}_{F}^{2},\;s.t.\;Z\geq 0,\;H\geq 0, \end{array}$$

where $X\in R^{m\times n}$ is the original non-negative matrix, $Z\in R^{m\times k}$ is the basis matrix, $H\in R^{k\times n}$ is the coefficient matrix, the rank k<min{m,n}, and the operator ${∥\cdot ∥}_{F}$ is the Frobenius norm. The multiplicative update rule [11] has been frequently used to minimize the objective function.

### 2.2. Joint NMF

jNMF is an advanced NMF model that decomposes multiple data into a common basis matrix and multiple coefficient matrices [4]. Given non-negative *I* matrices $X_{1}\in R^{m\times n_{1}}$, …, $X_{I}\in R^{m\times n_{I}}$, jNMF approximately decomposes with $X_{1}\approx Z^{+}H_{1}^{+}$, …, $X_{I}\approx Z^{+}H_{I}^{+}$ as the following objective function.

(2) $$\begin{array}{c} \begin{array}{c} \min_{Z,H_{1},\ldots ,H_{I}}\sum_{i=1}^{I}{∥X_{i}-ZH_{i}∥}_{F}^{2},\;\\ s.t.\;Z\geq 0,\;H_{i}\geq 0,i=1,\ldots ,I, \end{array} \end{array}$$

where $Z\in R^{m\times k}$ is a common basis matrix, $H_{i}\in R^{k\times n_{i}}$ is a *i*th coefficient matrix, and the rank $k<\min\{m,n_{1},\ldots ,n_{I}\}$. The basis matrix *Z* is a feature extracted matrix in which multiple data are integrated, and a coefficient matrix $H_{i}$ is a feature extracted matrix from each *i* dataset. It is useful for integrating heterogeneous data such as multi-omics data.

### 2.3. Semi-NMF

Semi-NMF [12] is a relaxed NMF algorithm that restricts only a non-negative constraint of the *H* matrix. Matrix *X* and matrix *Z* may have mixed signs. Semi-NMF decomposes with $X^{\pm }\approx Z^{\pm }H^{+}$, minimizing the following objective function.

(3) $$\begin{array}{c} \min_{Z,H}{∥X-ZH∥}_{F}^{2},\;s.t.\;H\geq 0, \end{array}$$

where $X\in R^{m\times n}$, $Z\in R^{m\times k}$, $H\in R^{k\times n}$, and the rank k<min{m,n}. From the perspective of clustering, we can also see the *Z* matrix as the cluster centroid, and the *H* matrix can be viewed as the cluster indicator.

### 2.4. Deep Semi-NMF

Deep semi-NMF [9] is a multi-layer feature extraction algorithm used to learn complex hierarchical representations of a dataset. Deep semi-NMF is a semi-NMF with a multi-layer concept that can learn hidden representations. Deep semi-NMF decomposes a given original *X* matrix into M+1 factors with $X^{\pm }\approx Z_{1}^{\pm }Z_{2}^{\pm }\ldots Z_{M}^{\pm }H_{M}^{+}$, minimizing the following objective function.

(4) $$\begin{array}{c} \begin{array}{c} \min_{Z_{m},H_{M}}{∥X-Z_{1}\left(Z_{2}\left(\cdots \left(Z_{M}H_{M}\right)\right)\right)∥}_{F}^{2},\;\\ s.t.\;H_{m}\geq 0,\;m=1,\ldots ,M, \end{array} \end{array}$$

where $H_{m}$ are restricted to be non-negative that are implicit representations of *m* layers as follows:

(5) $$\begin{array}{c} \begin{array}{c} H_{M-1}^{+}\approx Z_{M}^{\pm }H_{M}^{+},\\ \cdots \\ H_{2}^{+}\approx Z_{3}^{\pm }\cdots Z_{M}^{\pm }H_{M}^{+},\\ H_{1}^{+}\approx Z_{2}^{\pm }\cdots Z_{M}^{\pm }H_{M}^{+} \end{array} \end{array}$$

Implicit representations of layers are suitable clusters according to the corresponding attributes. The multi-layer model helps learning multiple low-dimensional representations of the original matrix.

## 3. Proposed Method

### 3.1. Joint Deep Semi-NMF

Our proposed model, JDSNMF, has advantages of non-linear dimensionality reduction and heterogeneous data integration. It adapts several advanced NMF approaches for feature extraction to learn complex non-linear manifolds from complex heterogeneous data. JDSNMF adapts joint NMF [4] to integrate multi heterogeneous data and adapts the multi-layer NMF principle and a non-linear activation function to represent non-linear manifolds. In addition, it uses a regularization term to prevent overfitting. Given matrices $X_{1}\in R^{C\times M_{1}}$, …, $X_{I}\in R^{C\times M_{I}}$, *I* is the number of multiple datasets, *C* is the number of samples (or features), and *M* is the number of individual features (or samples). JDSNMF decomposes the given original matrices by minimizing the following objective function.

(6) $$\begin{array}{c} \begin{array}{c} \min\sum_{i=1}^{I}{∥X_{i}-Ug\left(Zi_{1}g\left(Zi_{2}\ldots g\left(Zi_{N}Hi_{N}\right)\right)\right)∥}_{F}^{2}+\lambda {∥S∥}_{F}^{,}\\ s.t.\;Hi_{n-1}=g\left(Zi_{n}Hi_{n}\right),\;Hi_{0}\ldots Hi_{N-1}\geq 0,\\ S\in \{U,Zi_{1},\ldots ,Zi_{N},Hi_{N}\},\;n=1,\ldots ,N \end{array} \end{array}$$

where $U\in R^{C\times K_{0}}$ is a common sample (or feature) latent matrix, $Hi_{0}\in R^{K_{0}\times M_{i}}$ is a feature (or sample) latent matrix of the first layer, $Hi_{n}\in R^{K_{n}\times M_{i}}$ is a feature (or sample) latent matrix of a sublayer, and $Z_{n}\in R^{K_{n-1}\times K_{n}}$ is a junction latent matrix. The reduced dimension of the first layer is $K<\min\{C,M_{i}\}$, and the reduced dimension $K_{n}$ is smaller than the reduced dimension $K_{n-1}$ of the parent layer. JDSNMF uses a non-linear activation function *g* such as a sigmoid activation function and a ReLU activation function to make the decomposed *H* matrix non-negative with non-linearity.

### 3.2. Constructing Modules

The feature latent matrix and the sample latent matrix of the first layer are used in module construction. In the application of NMF, researchers have used two different approaches to construct modules. One is to assign each feature (or sample) to a module with a maximum value [13,14], and the other is to assign a set of significant features (or samples) from z-distribution to each module [4]. Because the distribution difference among modules may assign too many features and samples to specific modules in the former case, we used the latter case. In the sample latent matrix, samples larger than a predefined positive threshold (empirically set to μ+1.6449σ, 5%) were selected for each module. In the feature latent matrix, features larger than a given positive threshold and features smaller than a given negative threshold (empirically set to μ±2.5758σ, 1%) were selected for each module.

**Figure 2:**
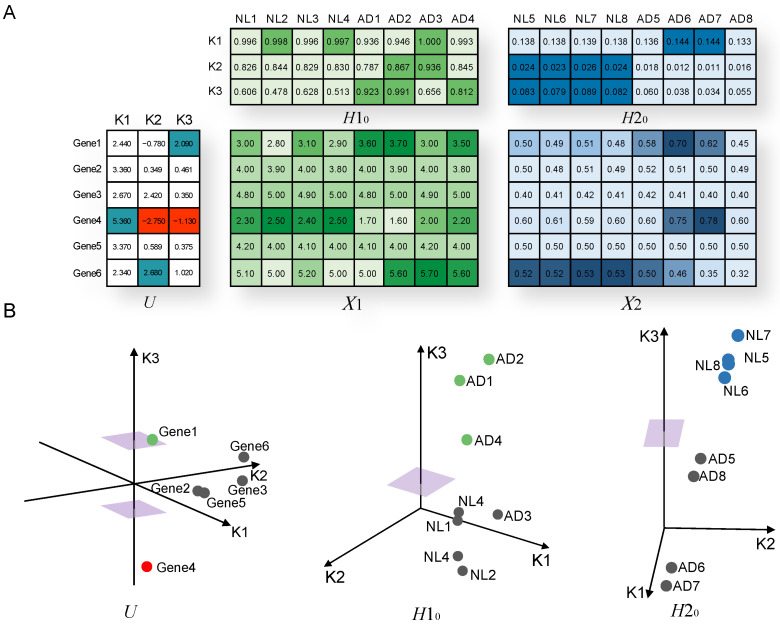
An example of the module construction. (**A**) Result of JDSNMF decomposition. In *U*, $H1_{0}$, and $H2_{0}$, cyan, green, and blue elements were larger than positive thresholds, and in *U*, $H1_{0}$, and $H2_{0}$, red elements were smaller than a negative threshold in *U*. (**B**) Genes and samples are coordinated for each axis, each of which represents a module. In *U*, $H1_{0}$, and $H2_{0}$, cyan, green, and blue elements are larger than positive thresholds, and in *U*, red elements are smaller than a negative threshold. Purple squares represent thresholds of the K3 module.

Figure 2 demonstrates a process of constructing modules using an example of feature-matched gene expression (GE) and DNA methylation (DM) data. In some AD samples, Gene 1 and Gene 6 were overexpressed and Gene 4 was underexpressed; Gene 1 and Gene 6 were hypermethylated and Gene 4 was hypomethylated. Figure 2A shows that GE and DM data were decomposed using the JDSNMF model. *U* is a feature latent matrix, and $H1_{0}$ and $H2_{0}$ are sample latent matrices of the first layer. Cyan, green, and blue elements in *U*, $H1_{0}$, and $H2_{0}$ are bigger than a positive threshold for each module, whereas red elements in *U* are smaller than a negative threshold for each module. We assumed that genes with values larger than a positive threshold (positive genes) increase GE or DM values of corresponding samples in the original data matrix and that genes with values less than a negative threshold (negative genes) decrease GE or DM values of corresponding samples in the original data matrix. In the K3 module, AD samples from the GE data and NL samples from the DM data were selected by thresholds; Gene 1 and Gene 4 are positive and negative genes, respectively. This result indicates that Gene 1 is more likely to be overexpressed and hypermethylated, and Gene 6 to be underexpressed and hypomethylated, in some of the AD samples. Figure 2B geometrically shows the process of K3 module construction. We coordinated genes and samples in three dimensions, each representing one module. The cyan circle, red circle, and purple squares indicate positive genes, negative genes, and thresholds for the K3 module, respectively.

## 4. Results

We conducted four experiments to validate the effectiveness of the proposed JDSNMF. First, we evaluated the feature extraction performance in the context of classification on sample-matched simulated data. Second, we evaluated the feature extraction performance on feature-matched multi-omics data.

The performance of JDSNMF using the GE data of the Addneuromed (ANM) and DM data of the MRC London Brainbank cohort as feature-matched data was compared with models using only GE data of the ANM cohort. We provide details of the comparison of the performances of AD/normal (NL) classification and mild cognitive impairment (MCI)/NL classification on the GE data of the ANM cohort. Third, we compared the performance of AD/NL classification according to the reduced dimension K on the Alzheimer’s Disease Neuroimaging Initiative (ADNI) cohort. Fourth, we highlight AD- and age-related modules and match the module to the known database, indicating that JDSNMF is effective in module analysis.

### 4.1. Dataset

We used a blood GE profile from the ANM consortium, a large cross-European AD biomarker study, and a follow-on Dementia Case Register (DCR) cohort in London. We downloaded the normalized RNA profile from the GEO: ANM Cohort 1 (GSE63060) and ANM Cohort 2 (GSE63061). The ANM Cohort 1 contains blood GE data of 145 patients with AD, 80 MCIs, and 104 NL, and the ANM Cohort 2 has those of 140 patients with AD, 109 MCIs, and 135 NLs after removing three borderline MCI samples and one other sample. We averaged the expression levels of probesets with the same RefSeq IDs, and then filtered out the uncommon RefSeq IDs between two cohorts (*n* = 20,017). We normalized ANM Cohorts 1 and 2 to reduce the batch effect using the Combat method from the ‘SVA’ R package [15].

We used a peripheral whole blood DM profile from the MRC London Brainbank for Neurodegenerative Disease [16]. We downloaded the DM profile from the GEO (GSE59685) that were converted to beta-values. The DM dataset had 80 subjects, some of which were annotated. We annotated 23 subjects that were not annotated in that study by the Braak stages [17,18] (i.e., Braak stages I and II—preclinical AD, Braak stages III and IV—prodromal AD, Braak stages V and VI—clinical AD). The DM data consisted of 68 patients with AD and 12 NLs. We mapped the CpG sites located within the promoter region, which is within a 200-base pair upstream region of the transcription start site (TSS200), to RefSeq IDs and averaged duplicated RefSeq IDs (*n* = 26,310). We filtered out the uncommon RefSeq IDs between the GE profiles and the DM profile. We also mapped RefSeq IDs to gene symbols and averaged the values of the same gene symbols in the GE and DM profiles (*n* = 12,011).

For the ADNI database, we downloaded robust multichip average normalized GE and raw DM profiles (IDAT format) from http://adni.loni.usc.edu (accessed on 1 September 2019). Both GE and DM profiles were obtained from whole blood. Because the GE profile did not contain diagnostic information, we obtained diagnosis of GE samples using the subject ID and visit code of the diagnostic information file (DXSUM_PDXCONV_ADNIALL.csv). The GE profile from ADNI consisted of 362 participants (NL: 246, AD: 116). We averaged the expression values of the same gene symbols (*n* = 18,727). Because ADNI has longitudinal data, there are samples with different diagnosis for each participant. Thus, we selected the DM samples examined on similar dates with GE samples for each participant. We obtained 294 participants (NL: 199, AD: 95), and we converted the IDAT file into a beta-value format using a “minifi” R package [19]. We mapped the CpG sites located within promoter regions (TSS200) to gene symbols. Then, we averaged β-values of the same gene symbols (*n* = 21,099). We filtered out the uncommon genes between the GE profiles and the DM profile (*n* = 15,922).

### 4.2. Hyperparameters and Settings for Experiments

In this section, the hyperparameters and settings of JDSNMF, NMF, deep semi-NMF, and classification models are described.

Our JDSNMF model has four key hyperparameters: the number of layers, the reduced dimensions of each layer, a layer to use for classification, and an L2 norm parameter. In the NMF method, the hyperparameters were the updated algorithm, the number of reduced dimensions, and the initialization method. The hyperparameters in the deep semi-NMF method were the same as those in the JDSNMF model.

For JDSNMF and deep semi-NMF, the Adam Optimizer [20] and early stopping were used. We used a sigmoid function as an activity function to make the decomposed sample latent matrix non-negative with non-linearity. The initialization strategy for a basis matrix and a coefficient matrix is an important step that affects the results. We used singular value decomposition (SVD), a popular initialization strategy in NMF tasks [21]. In a study by [22] using collective deep matrix factorization, SVD performed best when initializing each matrix to be decomposed.

We used an NMF method in the scikit-learn library [23] and implemented the deep semi-NMF method ourselves using Tensorflow [24]. JDSNMF is implemented using Tensorflow and code is available at https://github.com/dmcb-gist/Joint-deep-semi-NMF, accessed on 30 January 2020. The scikit-learn python library [23] for the linear support vector machine (SVM) and random forest (RF) based on 10,000 trees was used. For the deep neural network (DNN), the Tensorflow library [24] was used. For the DNN, we set three fully connected layers with L2 regularization and early stopping.

### 4.3. Sample-Matched Simulated Dataset Experiments

We evaluate the robustness performance and feature extraction performance of our proposed JDSNMF model on sample-matched simulated datasets.

Three datasets with small sample sizes and high dimensionality are simulated, while most of the features are irrelevant to the objective. We generated the predictor matrix $X\in R^{S\times F}$, drawn from a standard normal distribution, where S is the number of samples and F is the number of features. We set $x_{sj}$ as the *s*th sample and *j*th feature. The class label probability $y_{s}$ is generated by the logistic regression model as follows [25]:

(7) $$\begin{array}{c} \begin{array}{c} y_{s}=\frac{exp\left(\beta_{0}\;+\;\sum_{f=1}^{F}\beta_{f}x_{sf}\;+\;\epsilon \right)}{\;1\;+\;exp\left(\beta_{0}\;+\;\sum_{f=1}^{F}\beta_{f}x_{sf}\;+\;\epsilon \right)}, \end{array} \end{array}$$

where $\beta_{f}$ represents the coefficient of *j*th feature, and ϵ represents random error generated by N(0,1.6). Three simulated data are generated by the above procedure. We set 20 nonzero coefficient β and 980 zero coefficient β. The value of nonzero coefficient is from {±10}. The nonzero coefficient features represent core features of three simulated data. We also choose the sample such that the class labels are consistent across three simulated data. We set training sample size as 750, validation sample size as 50, and test sample size as 100.

**Figure 3:**
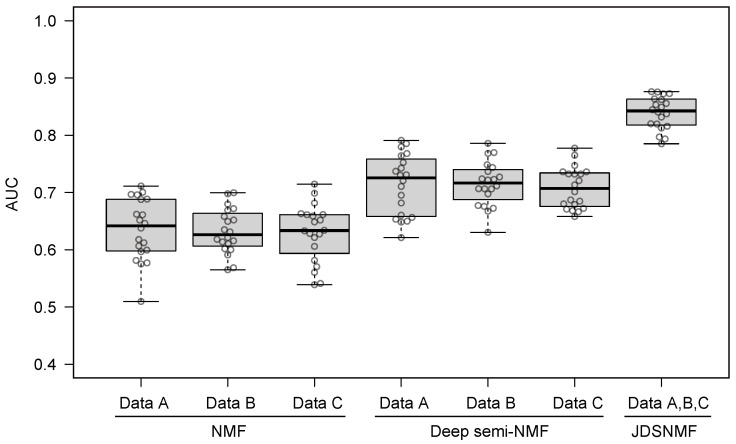
Test AUC comparison of NMF, deep semi-NMF, and JDSNMF on simulated data.

We evaluate the feature extraction performance with NMF, deep semi-NMF, and JDSNMF on simulated data (A,B,C). We measure the classification performance of the output from each feature extraction model using SVM. The optimal hyperparameters of each feature extraction model are searched by validation data. The hyperparameters of SVM were fixed. We repeated the analysis 20 times with different simulated datasets. Figure 3 shows the box plot analysis of the area under the curve (AUC) from test sets. We can easily show that integrating multi-omics data using JDSNMF improves the performance. We also observed that deep semi-NMF outperformed NMF. These results suggest that non-linearity and integration of multiple data are required to improve classification performance and that more important information for classification is extracted.

### 4.4. Feature-Matched Real Dataset Experiments

To evaluate our model’s ability to identify latent information predictive of AD from feature-matched omics data, we compared it with a feature selection approach based on differentially expressed genes, NMF, and a deep semi-NMF. The GE data of the ANM cohort were used for all models. Additionally, the DM data of the MRC London Brainbank cohort were used for JDSNMF.

Five-fold cross validation (CV) was employed to evaluate performance. In AD/NL classification, we divided samples into training, validation, and test sets (3:1:1 ratio) in each fold. The optimal hyperparameters were searched by validation data. In MCI/NL classification, we used optimal hyperparameter sets that had the best performance in the AD/NL classifications (see Supplementary Table S1 and the “Hyperparameters in MCI/NL classification on Addneuromed cohort ” section in the Additional Files for details). For the feature selection method, differentially expressed genes were selected using a training set with the “lmFit” function of the limma R package [26]. Statistically significant genes were obtained based on the adjusted *p*-value. For the baseline, we randomly selected the same number of genes selected in the feature selection model (an adjusted *p*-values < 5.00 × $10^{-4}$) in each CV fold. We measured the classification performance of the output from each model using DNN, SVM, and RF. The hyperparameters of DNN, SVM, and RF were fixed.

**Figure 4:**
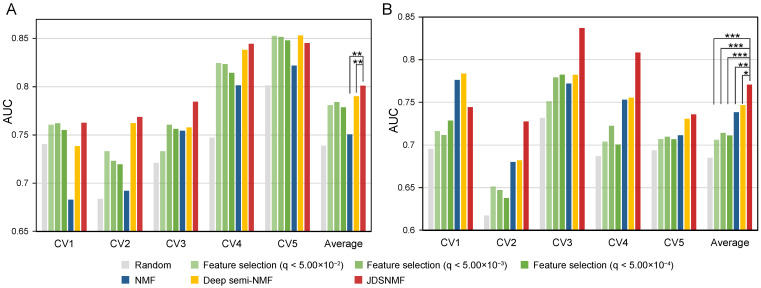
Comparison of the feature selection methods, NMF, deep semi-NMF, and JDSNMF, using the Addneuromed cohort. (**A**) AD/NL classification. (**B**) MCI/NL classification. Three classifiers (deep neural network, support vector machine, and random forest) were used as classifiers, and area under the curve (AUC) values between true positives and false positive rates were measured for classification performance. Then, the average AUC values of the three classifiers are shown for each CV fold. Stars indicate statistical significance with a Wilcoxon signed-rank test (*, *p*-value < 0.1; **, *p*-value < 0.05; ***, *p*-value < 0.01).

Figure 4 shows the averages of AUC values between true and false positives of three classifiers for each CV fold. The classification performances of each of the three classifiers are shown in Supplementary Figure S1. JDSNMF outperformed the other models in the classification of AD and NL (the average AUC of the three classifiers was 0.801) and in the classification of MCI and NL (the average AUC of the three classifiers was 0.771). When we applied a Wilcoxon signed-rank test for 15 AUC values (5 CVs × 3 classifiers), JDSNMF statistically significantly outperformed NMF and deep semi-NMF for the AD/NL classification and statistically significantly outperformed feature selection methods, NMF, and deep semi-NMF for MCI/NL classification. In the comparison of validation sets using various hyperparameters, we also confirmed that JDSNMF performed better in most situations (Supplementary Figures S2–S4). These results suggest that the DM data of other cohorts were helpful in extracting latent features and that JDSNMF is applicable to feature-matched dataset experiments as well.

### 4.5. Classification Performance Comparison According to the Reduced Dimension K on ADNI Cohort

The reduced dimension K is a critical hyperparameter in all matrix factorization models. If K is set too small, the data do not fit well to the model. On the other hand, if K is too large, overfitting occurs. In our experiments, K is determined through validation data. To further demonstrate the effects of K on the performance of JDSNMF, we compared the performance according to K. The classification performance is the average of the performances of the DNN, SVM, and RF models. The hyperparameters of DNN, SVM, and RF were fixed.

**Figure 5:**
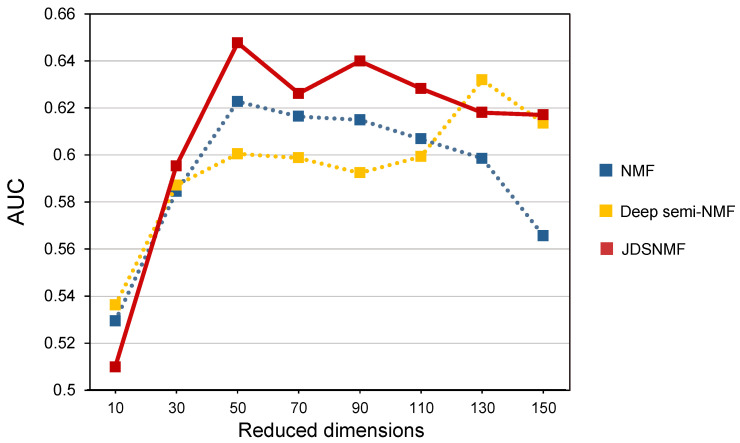
Comparison of classification performance on the Alzheimer’s Disease Neuroimaging Initiative cohort. Average area under the curve (AUC) values of SVM, RF, and DNN for AD/NL classification by each model with respect to reduced dimensions.

Figure 5 shows the classification performance where K varies from 10 to 150 for NMF, deep semi-NMF, and JDSNMF on the ADNI cohort. We observed that K did influence the classification performance of JDSNMF. However, JDSNMF was consistently outperformed by existing methods under a wide range of K values. In all K except for dimensions 10 and 130, the JDSNMF model outperformed other models. When we applied a Wilcoxon signed-rank test for 24 AUC values (8 reduced dimensions × 3 classifiers), JDSNMF statistically significantly outperformed deep semi-NMF (*p*-value < 0.05). The classification performances of each of the three classifiers are shown in Supplementary Figure S5.

### 4.6. Module Analysis

In this section, we attempt to interpret the clinical and biological meanings of the latent matrices from the GE data of the ANM cohort and the DM data of the MRC London Brainbank cohort. We identified AD- and age-related modules and matched the module to the known database. We used the GE sample latent matrix, DM sample latent matrix, and feature latent matrix from the JDSNMF model with the best performance in the “Feature-matched real dataset experiments” section (number of layers, three; reduced dimensions of each layer, 60, 59, 44; layer used for classification, third layer; and L2 norm parameter, 0.01). We constructed the 60 modules from the GE sample latent matrix, DM sample latent matrix, and feature latent matrix.

#### 4.6.1. AD-Related Module

We used a locally interpretable model-agnostic explanation (LIME) [27] as the xAI method to identify the most important AD-related module in AD/NL classification. LIME is a model-agnostic technique that interprets complex models by approximating them locally. LIME learns to understand a black-box classifier by perturbing the input data and interpreting changes in prediction. We used LIME to locally approximate SVM, RF, and DNN models to the simple linear models to understand the models and identify the module that is important for AD classification. In addition, we calculated the relative importance of each module using RF.

Module 7 had the highest sum of LIME values for the three classifiers (Supplementary Figure S6). At the same time, module 7 was also the most important module for classification according to RF importance feature measures. Samples and significant genes included in module 7 are shown in Supplementary Figures S7 and S8. We performed a functional enrichment test of module 7 with KEGG pathways [28] using enrichR [29,30]. Table 1 shows the significantly enriched pathways (*q*-value < 0.0001) in module 7. Among them, the “Alzheimer disease” pathway was statistically significant (*q*-value = 9.00 × $10^{-5}$). In detail, 15 genes in module 7 overlapped with genes in the AD pathway. Among them, 3 (*ATP5F1A*, *TNFRSF1A*, and *SNCA*) were positive genes and 12 were negative genes (*ATP5PF*, *ATP5PO*, *COX6A1*, *COX6C*, *COX7B*, *COX7C*, *NDUFB3*, *NDUFB2*, *NDUFA1*, *NDUFS5*, *NDUFS4*, and *UQCRQ*). We surveyed these genes from previous studies for relevance to AD. SNCA (α-synuclein) is widely known to play a role in the pathogenesis of AD. In a recent paper, elevated mRNA expression and low methylation of SNCA were observed in AD [31]. The samples included in module 7 showed similar results, with 12 AD samples and 1 NL sample in GE and 2 AD samples and 1 NL sample in DM. SNCA mRNA expression values of the module 7 samples were significantly higher than those of the rest of the samples (*p*-value of the two tailed *t*-test = 1.49 $\times \;10^{-2}$). SNCA methylation β-values of module 7 samples were significantly lower than those of the rest of the samples (*p*-value of the two tailed *t*-test = 2.40 $\times \;10^{-6}$). TNFRSF1A (TNF Receptor Superfamily Member 1A), known as TNFR1, may lead to neurotoxicity and mediates the A β -induced caspase-dependent death pathway in neuronal cells [32]. In a previous study [33], although the protein level of TNFR1 was increased in the neurons of patients with AD, no significant differences in TNFR1 mRNA expression were identified using in situ hybridization. However, we observed that TNFR1 mRNA expression levels of the module 7 samples (12 AD and 1 NL samples) were significantly higher than those of the rest of the samples (*p*-value of the two tailed *t*-test = 4.30 $\times \;10^{-4}$). As the previous study analyzed frontal cortex samples of only 12 AD and 12 NL cases, it is probable that TNFR1 mRNA expression levels would have differentially increased in a subgroup of Alzheimer’s patients. The *ATP5F1A* gene, among the positive genes, and the 12 negative genes are translated into subunits of ATP synthase, cytochrome c oxidase (COX), NADH-ubiquinone oxidoreductase, and ubiquinol-cytochrome c oxidoreductase, which are components of the mitochondrial respiratory chain. Previous studies reported that mitochondrial dysfunction is related to AD [34]. Reduced COX expression in the AD brain was observed in another study [35].

Other pathways are known to be directly or indirectly related to AD. For example, differential expression of oxidative phosphorylation genes is observed in AD [29]. In wild-type mice, non-alcoholic fatty liver disease has been shown to cause AD [36]. A relationship between natural killer cells and AD was also reported [37,38].

**Figure 6:**
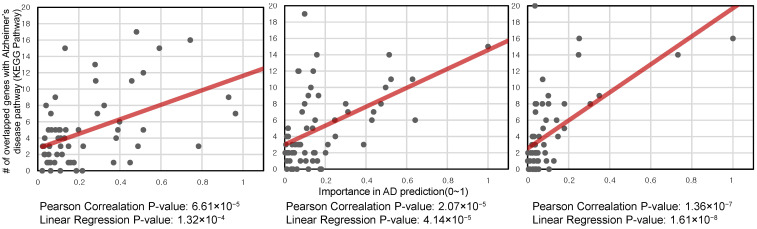
Relationship between the genes overlapping with Alzheimer’s disease (AD) KEGG pathway and importance in AD prediction. For each module, linear regression was performed, and the Pearson correlation was calculated for the relationship between the number of genes overlapping with the AD KEGG pathway and LIME importance for AD prediction. Three examples with different hyperparameters are shown: the best performing two-layer model (**left**), the best performing three-layer model (**middle**), and the best performing four-layer model (**right**) in the AD/NL classification task on the Addneuromed cohort.

For each module, we performed linear regression analysis and calculated the Pearson correlation coefficient between the LIME importance score in the AD classification and the number of genes overlapped with the AD pathway in KEGG pathway analysis. Figure 6 shows the results for three example models: the best performing two-layer, three-layer, and four-layer models in the “Classification performance comparison on Addneuromed cohort” section (see Supplementary Figures S6, S9, and S10 for detail). In all three cases, there was a statistically significant relationship between importance in the AD prediction and the number of genes overlapped with the AD pathway.

#### 4.6.2. Age-Related Module

To study other modules further, we used age information and found age-related modules by applying the following linear regression model to the GE and DM sample latent matrix.

(8) $$\begin{array}{c} Y_{ij}=\beta_{j}+\gamma_{j}Age_{i}+\delta_{j}Sex_{i}+\mu_{j}Diagnosis_{i}+\sum_{k=1}^{N}\alpha_{jk}PC_{ik}, \end{array}$$

where $Y_{ij}$ is the sample latent matrix value corresponding to sample *i* in module *j*, $Age_{i}$ is the age of the sample *i* with coefficient $\gamma_{j}$, $Sex_{i}$ is the sex of the sample *i* with coefficient $\delta_{j}$, $Diagnosis_{i}$ is the AD state of the sample *i* with coefficient $\delta_{j}$, and $PC_{ik}$ denotes the value of the *k*-th principal component of the sample latent matrix for the *i*-th sample with coefficient $\alpha_{ij}$. *N* is the number of PCs that are not related to age. $\beta_{j}$ is an intercept for regression. We identified a relation with age for each module from $\gamma_{j}$. As a result, modules 1, 5, and 22 were statistically significantly related to age (Supplementary Figure S11). We focused on module 1, the most statistically significant (*p*-value = 4.58 $\times \;10^{-3}$ and 3.53 $\times \;10^{-2}$ for GE and DM, respectively). In particular, $\gamma_{j}$ was positive for GE, indicating that GE values of genes in module 1 were positively correlated with age. In contrast, $\gamma_{j}$ was negative for DM, indicating that DM values of genes in module 1 were negatively correlated with age.

We overlapped the genes included in module 1 with gene ontology terms [39] and KEGG pathways using enrichR. A total of 34 GO terms and 44 pathways were significantly enriched (*q*-value < 0.05) in module 1 (Supplementary Table S2). The top terms in the list, such as “neutrophil-mediated immunity” (*q*-value: 2.51 $\times \;10^{-14}$), “neutrophil activation involved in immune response” (*q*-value = 2.9 $\times \;10^{-14}$), and “neutrophil activation involved in immune response” (*q*-value = 4.49 $\times \;10^{-14}$) are related to innate immunity functions of neutrophils. It is well known that the aging process deteriorates neutrophil functions such as phagocytic capacity, degranulation, and superoxide generation [40]. “Longevity regulating pathway” (*q*-value = 1.31 $\times \;10^{-2}$) is also significantly enriched, which is related to the aging process.

In addition, when we examined the overlaps between 240 genes in module 1 and 305 aging-related genes from the GenAge database [41] with Fisher’s exact test, the *p*-value was 9.99 $\times \;10^{-3}$, showing the significance of the overlaps.

### 4.7. Experiments on Bioimaging Data

As brain imaging data are also important for the diagnosis of AD, we evaluated the feature extraction performance of our proposed JDSNMF model on sample-matched bioimaging datasets. The experiments were performed on magnetic resonance imaging (MRI) regions of interest (ROIs) and AV-45 amyloid positron emission tomography (PET) ROI datasets from ADNI-TADPOLE ( http://adni.loni.usc.edu, accessed on 13 July 2021). We selected 750 ADNI2 samples with both data types. The numbers of NL, significant memory concern (SMC), early MCI (EMCI), late MCI (LMCI), and AD are 180, 102, 170, 156, and 142, respectively.

We measured the classification performance of extracted features from each feature extraction model using SVM. The reduced dimension *K* of each model was selected by a validation set from each fold. The AUC values for one-vs-rest were calculated for multiclass classification. Table 2 shows the average AUC values of five-fold CV for NL/MCI, MCI/AD, NL/AD, and NL/SMC/EMCI/LMCI/AD classification tasks. JDSNMF had higher AUC values than others in the MCI/AD classification task. In the NL/AD and NL/SMC/EMCI/LMCI/AD classification tasks, JDSNMF had similar performance to or slightly higher than PET-based joint deep semi-NMF. We also observed that deep semi-NMF outperformed NMF on these three classification tasks. However, the performance of the non-linearity based models were worse than that of NMF in NL/MCI classification. Taken together, these results suggest that our model is generally applicable for various data types.

## 5. Discussion and Conclusions

We introduced a novel JDSNMF model—a non-linear feature extraction model—that can capture shared latent representations from complex multi-omics data. The proposed model is applicable to the existing sample-matched multi-omics integration and the new feature-matched multi-omics integration. JDSNMF demonstrated significant improvements in multi-omics feature extraction tasks on sample-matched simulated data and feature-matched multi-omics data. In AD experiments, we identified AD-related and age-related modules through the recent xAI model and traditional regression approaches. We observed that many AD-related genes were included in modules important for AD prediction and that genes in the age-related modules were significantly enriched with known aging-related genes. These results suggest that it is applicable not only to analysis of large cohorts with multi-omics data but also to integration analysis between single omics cohorts. Furthermore, feature-matched integration analysis can be applied to cross-species comparison, cross-disease comparison, and cross-cohort comparison. We also confirmed that our model is applicable to bioimaging data by further experiments.

However, this method has some limitations. Our model involves simple multi-task learning with uniform loss weights. This can lead to an optimization conflict while learning to reconstruct each omic datum. In the future, we will apply advanced multi-task learning methods [42] to the JDSNMF method.

## Figures and Tables

**Table 1:** Enriched KEGG pathways of the module 7.

KEGG Pathways	Overlap	*q*-Value
Ribosome	23/153	7.35 × $10^{-11}$
Oxidative phosphorylation	19/133	1.13 × $10^{-8}$
Parkinson’s disease	18/142	1.75 × $10^{-7}$
Phagosome	18/152	3.96 × $10^{-7}$
Influenza A	19/171	3.46 × $10^{-7}$
Thermogenesis	21/231	1.65 × $10^{-6}$
Viral myocarditis	11/59	1.99 × $10^{-6}$
Graft-versus-host disease	9/41	6.75 × $10^{-6}$
Non-alcoholic fatty liver disease (NAFLD)	15/149	2.83 × $10^{-5}$
Leukocyte transendothelial migration	13/112	2.73 × $10^{-5}$
Natural killer cell-mediated cytotoxicity	14/131	2.61 × $10^{-5}$
Toxoplasmosis	13/113	2.52 × $10^{-5}$
Tuberculosis	16/179	4.18 × $10^{-5}$
Pathogenic Escherichia coli infection	9/55	5.36 × $10^{-5}$
Leishmaniasis	10/74	8.38 × $10^{-5}$
Alzheimer disease	15/171	9.00 × $10^{-5}$

**Table 2:** Performance comparison of NMF, deep semi-NMF, and JDSNMF on bioimaging data. Bold text indicates the best performance.

Tasks	NMF		Deep semi-NMF		JDSNMF
	MRI	PET	MRI	PET	MRI, PET
NL/MCI	0.630	**0.703**	0.569	0.696	0.665
MCI/AD	0.831	0.811	0.838	0.826	**0.843**
NL/AD	0.935	0.963	0.949	0.972	**0.973**
NL/SMC/EMCI /LMCI/AD	0.624	0.677	0.678	0.717	**0.718**

## Data Availability

Genetic data and bioimaging data used in this article were obtained from the Alzheimer’s disease Neuroimaging Initiative (ADNI) database https://ida.loni.usc.edu (accessed on 1 September 2019 and 13 July 2021, respectively). JDSNMF is publicly available at https://github.com/dmcb-gist/Joint-deep-semi-NMF (accessed on 30 January 2020).

## Supplementary Materials

The following figures and tables are available online at https://www.mdpi.com/article/10.3390/jpm11080686/s1. Table S1: Top AUC scores of validation sets with various hyper-parameters in Addneuromed cohort. Figure S1: Comparison of the feature selection method, joint deep semi-NMF (JDSNMF) model, and other dimension reduction models using three classifiers on the Addneuromed cohort. Figure S2: Average AUC of validation sets using the two-layer model with hyperparameters (a layer used for classification and the number of reduced dimensions) on the Addneuromed cohort. Figure S3: Average AUC of validation sets using the three-layer model with hyperparameters (a layer used for classification and the number of reduced dimensions) on the Addneuromed cohort. Figure S4: Average AUCs of validation sets using the four-layer model with hyperparameters (a layer to use for classification and the number of reduced dimensions) on the Addneuromed cohort. Figure S5: Classification performance of the feature selection method, joint deep semi-NMF model (JDSNMF), and other dimension reduction models using three classifiers on the ADNI cohort. Figure S6: Identification of AD-related modules using the best performing three-layer model on the ANM validation set. Figure S7: Top 10 positive genes and negative genes in the AD-related module. Figure S8: Samples larger than a predefined positive threshold (empirically set to μ + 1.6449σ) in the AD-related module (module 7 of the best performing model in the ANM validation set). Figure S9: Identification of the AD-related module using best performing two-layer model in the ANM validation set. Figure S10: Identification of AD-related modules using the best performing four-layer model in the ANM validation set. Figure S11: Identification of modules related to age. Table S2: Significantly enriched gene ontology terms and KEGG pathways of genes in module 1.
