# Supplementary material for: JDSNMF: Joint Deep Semi-Non-Negative Matrix Factorization for Learning Integrative Representation of Molecular Signals in Alzheimer’s Disease
Source: J Pers Med. 2021 Jul 21;11(8):686. doi: 10.3390/jpm11080686 (PMC8400727; doi:10.3390/jpm11080686)
Supplement: Supplementary file 1 [file jpm-11-00686-s001.zip › jpm-1265691-supplementary.pdf]

Supplementary Materials

JDSNMF: joint deep semi-non-negative matrix factorization for learning integrative representation of molecular signals on Alzheimer's disease

Sehwan Moon and Hyunju Lee*

[1]School of Electrical Engineering and Computer Science, Gwangju Institute of Science and Technology, Gwangju, South Korea.

* hyunjulee@gist.ac.kr

Hyperparameter tuning in AD/NL classification on Addneuromed cohort.

Our joint deep semi-non-negative matrix factorization method (JDSNMF) model and deep semi-NMF model have four key hyperparameters: the number of layers, the reduced dimensions of each layer, a layer to use for classification, and a L2 norm parameter. In Addneuromed (ANM) cohort, we changed the first layer dimension of JDSNMF model from 20 to 79, because it should be smaller than the sample size of methylation data (80). We varied the first layer dimension of deep semi-NMF model from 20 to 120. In the second layer model, we changed the dimension by 1/5, 2/5, 3/5, 4/5, and 1 of the upper layer. In the third layer model, we varied the dimension by 1/4, 2/4, 3/4, and 1 of the upper layer. In the fourth layer model, we changed the dimension by 1/3, 2/3, and 1 of the upper layer. We tried L2 regularization values with 0.0001, 0.001, and 0.01.

Hyperparameters in MCI/NL classification on Addneuromed cohort.

In the classification of MCI and NL, we used each model with hyperparameters sets that had the best performance from the AD and NL classifications. The best performing hyperparameters were selected as follows (see Supplementary Table S1 for detail): the number of layers, the reduced dimensions of each layer, the layer for classification, and the L2 norm parameter were three layers, (60, 59, 44), the third layer, and 0.01, respectively, for JDSNMF, and four layers, (60, 59, 39, 38), the first layer, and 0.01, respectively, for deep semi-NMF model. InNMF model, hyperparameters with the best performance from the five-fold CV sets were coordinate descent solver, 90 dimensions, random initialization for the update algorithm, the number of reduced dimension, and the initialization method, respectively.

Comparison of classification performance according to hyperparameters.

Figures S1, S2, and S3 shows that comparison of performance by varying the reduced dimension in the two-layer model, the three-layer model, and the four-layer model in the ANM cohort, respectively. In ANM, we observed a slight tendency for performance to improve as dimension increased. In most cases, we observed that the performance of JDSNMF is high.

	Layer to use for classification	Reduced dimensions of 1st layer	Reduced dimensions of 2nd layer	Reduced dimensions of 3rd layer	Reduced dimensions of 4th layer	λ (L2 Norm)	Fold 1	Fold 2	Fold 3	Fold 4	Fold 5	Mean
1	3rd layer	60	59	44		0.01	0.8823	0.7912	0.8196	0.7826	0.8795	0.8310
2	2nd layer	60	47			0.0001	0.8955	0.7923	0.8056	0.7982	0.8605	0.8304
3	2nd layer	60	59			0.0001	0.8953	0.7805	0.8143	0.7973	0.8601	0.8295
4	2nd layer	60	35			0.001	0.8894	0.7820	0.8307	0.7924	0.8390	0.8267
5	2nd layer	60	59			0.001	0.8967	0.7791	0.8133	0.7841	0.8598	0.8266
6	2nd layer	60	35			0.0001	0.8918	0.7783	0.8273	0.7956	0.8355	0.8257
7	2nd layer	40	39			0.0001	0.8879	0.7702	0.8051	0.7928	0.8723	0.8257
8	2nd layer	79	78	19		0.01	0.8839	0.7402	0.8441	0.7720	0.8808	0.8242
9	3rd layer	40	39	38		0.001	0.8690	0.7883	0.8017	0.8082	0.8534	0.8241
10	2nd layer	79	47			0.01	0.8924	0.7653	0.8060	0.7983	0.8566	0.8237
11	2nd layer	40	39			0.001	0.8851	0.7671	0.8036	0.7926	0.8632	0.8223
12	3rd layer	79	39	38		0.0001	0.8733	0.7696	0.7912	0.7963	0.8775	0.8216
13	3rd layer	60	44	43		0.01	0.8812	0.7702	0.8101	0.7731	0.8711	0.8211
14	3rd layer	79	78	58		0.001	0.8791	0.7772	0.8248	0.7825	0.8416	0.8210
15	2nd layer	60	35			0.01	0.8848	0.7754	0.8111	0.7886	0.8444	0.8209
16	3rd layer	79	78	77		0.01	0.8740	0.7878	0.8170	0.7623	0.8632	0.8209
17	2nd layer	60	44	43		0.01	0.8731	0.7599	0.8293	0.7660	0.8758	0.8208
18	2nd layer	60	59			0.01	0.8835	0.7788	0.8010	0.7686	0.8715	0.8207
19	2nd layer	40	39	38		0.001	0.8577	0.7717	0.8046	0.8014	0.8679	0.8207
20	2nd layer	79	62			0.01	0.8813	0.7858	0.7857	0.7904	0.8602	0.8207
21	2nd layer	60	47			0.001	0.8939	0.7837	0.8094	0.7918	0.8221	0.8202
22	3rd layer	79	39	38		0.01	0.8786	0.7611	0.8051	0.7794	0.8767	0.8202
23	2nd layer	60	47			0.01	0.8726	0.7851	0.7943	0.7801	0.8679	0.8200
24	2nd layer	60	59	44		0.0001	0.8912	0.7568	0.8030	0.7713	0.8739	0.8192
25	3rd layer	40	39	38		0.01	0.8591	0.7935	0.8000	0.7765	0.8639	0.8186
26	2nd layer	79	47			0.0001	0.8760	0.7765	0.7869	0.7951	0.8572	0.8184
27	2nd layer	79	78	77		0.01	0.8839	0.7754	0.7904	0.7761	0.8636	0.8179
28	1st layer	79	78	19		0.01	0.8776	0.7678	0.8171	0.7790	0.8480	0.8179
29	3rd layer	79	58	57		0.01	0.8733	0.7728	0.8120	0.7750	0.8550	0.8176
30	2nd layer	79	58	57		0.001	0.8787	0.7581	0.8144	0.7799	0.8565	0.8175
31	3rd layer	79	78	77	76	0.01	0.8774	0.7673	0.7968	0.7775	0.8684	0.8175
32	3rd layer	60	59	44		0.0001	0.8613	0.7997	0.7725	0.7957	0.8581	0.8175
33	3rd layer	60	59	44		0.001	0.8722	0.7727	0.8035	0.7585	0.8774	0.8168
34	3rd layer	60	59	58		0.01	0.8780	0.7487	0.8122	0.7876	0.8562	0.8165
35	2nd layer	60	59	29		0.01	0.8758	0.7659	0.7832	0.8212	0.8365	0.8165
36	2nd layer	79	62			0.001	0.8730	0.7894	0.7759	0.7922	0.8463	0.8153
37	2nd layer	40	39	38		0.0001	0.8739	0.7851	0.7899	0.7949	0.8326	0.8153
38	3rd layer	79	78	77		0.001	0.8688	0.7680	0.8259	0.7626	0.8498	0.8150
39	2nd layer	79	78			0.01	0.8919	0.7710	0.7855	0.7799	0.8465	0.8150
40	2nd layer	60	59	44		0.01	0.8818	0.7655	0.7943	0.7705	0.8602	0.8145

Table S1: Top AUC scores of validation sets with various hyper-parameters in Addneuromed cohort.

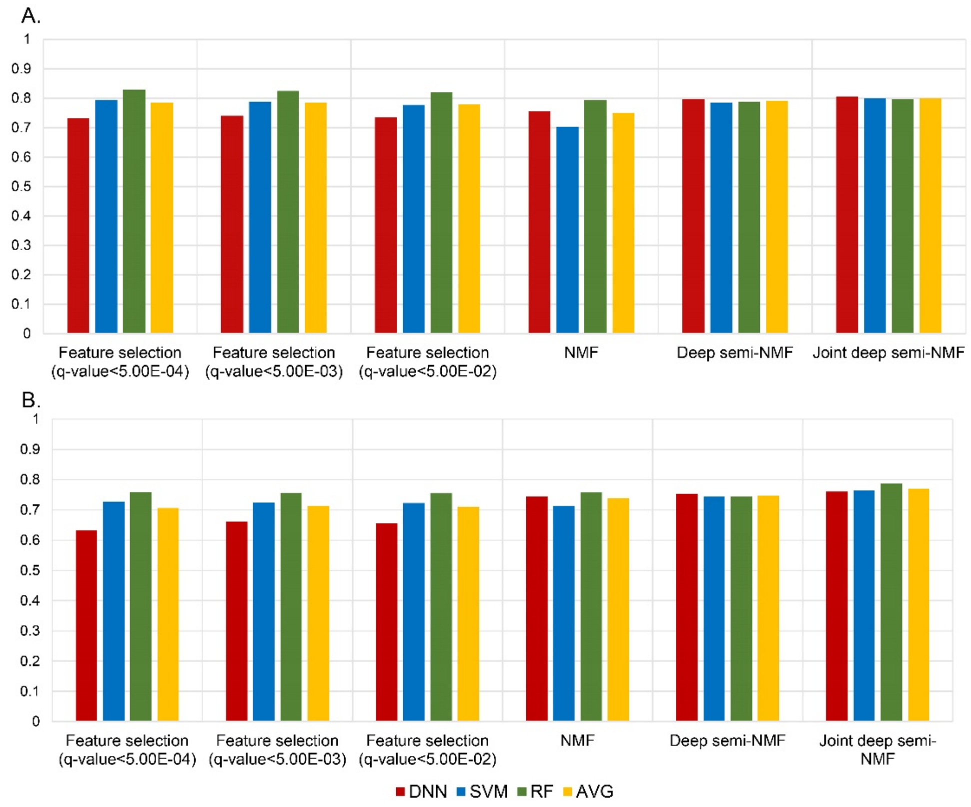

Figure S1: Comparison of the feature selection method, joint deep semi-NMF (JDSNMF) model, and other dimension reduction models using three classifiers for the Addneuromed cohort. (A) AD/NL classification. (B) MCI/NL classification. Deep neural network (DNN), support vector machine (SVM), and random forest (RF) were used and area under the curve (AUC) between true positives and false positive rates were measured for classification performance. Red, blue, and green bars show the performance using DNN, SVM, and RF, respectively. Yellow bars show the average AUC of the three classifiers.

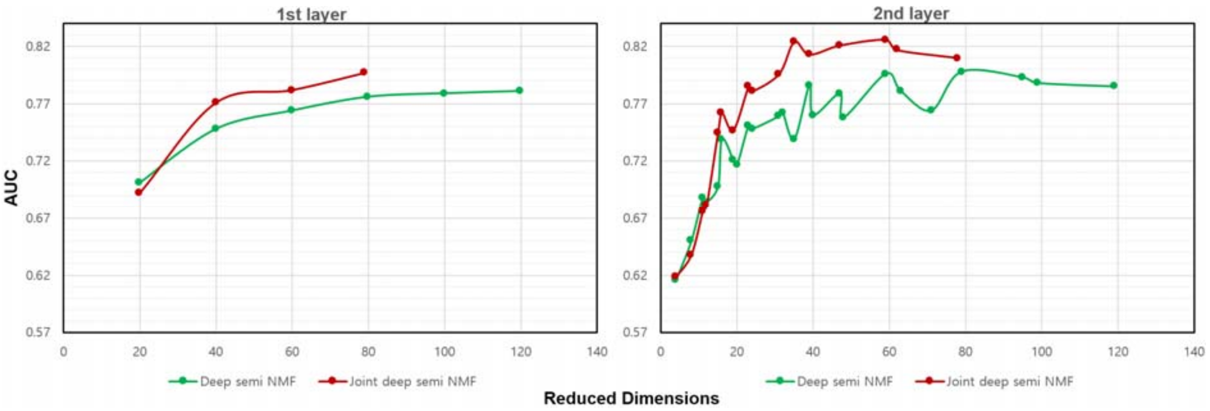

Figure S2: The average AUC of validation sets using the two-layer model according to hyper-parameters (a layer used for classification and the number of reduced dimensions) on Addneuromed cohort.

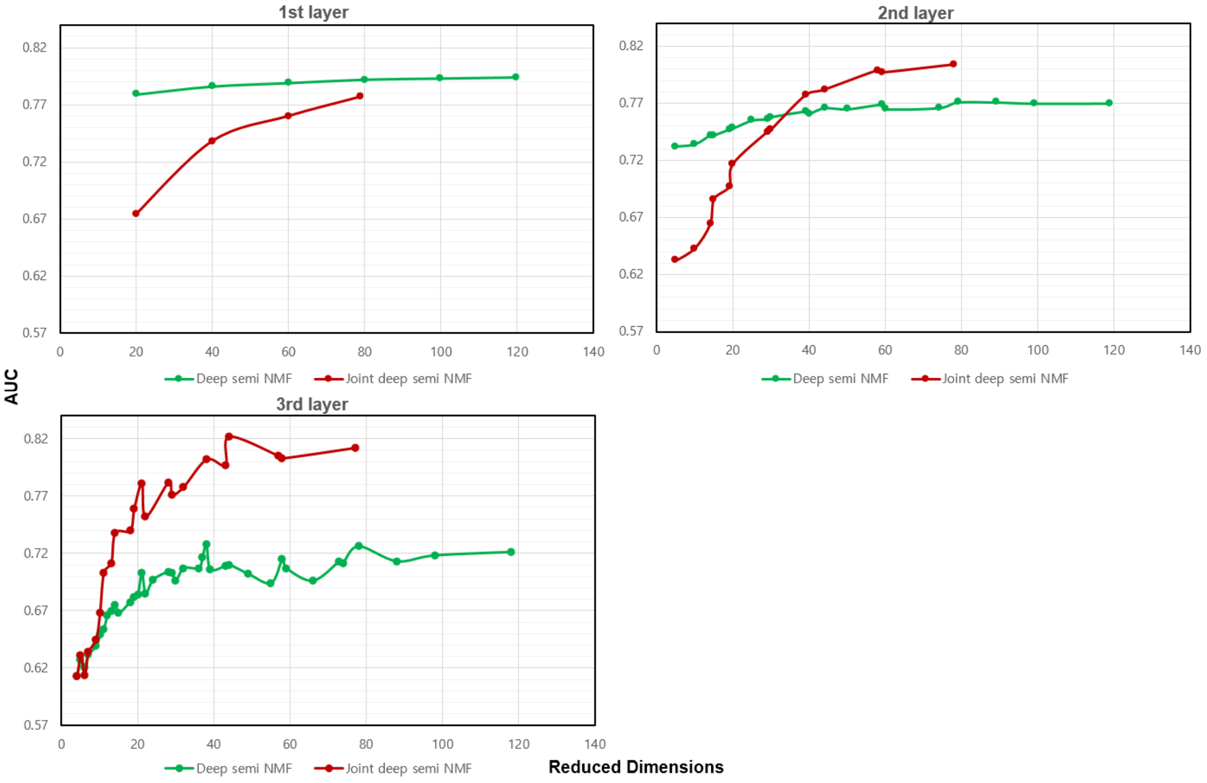

Figure S3: The average AUC of validation sets using the three-layer model according to hyper-parameters (a layer used for classification and the number of reduced dimensions) on the Addneuromed cohort.

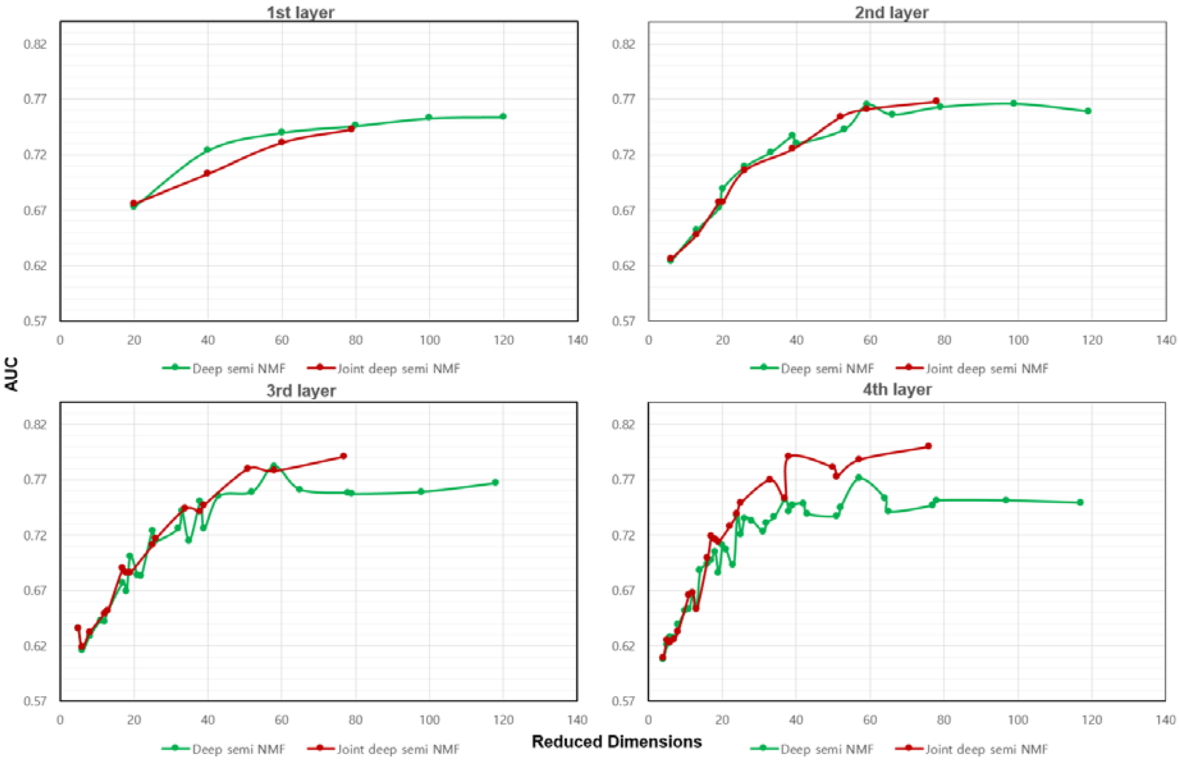

Figure S4: The average AUCs of validation sets using the four-layer model according to hyperparameters (a layer to use for classification and the number of reduced dimensions) on Addneuromed cohort.

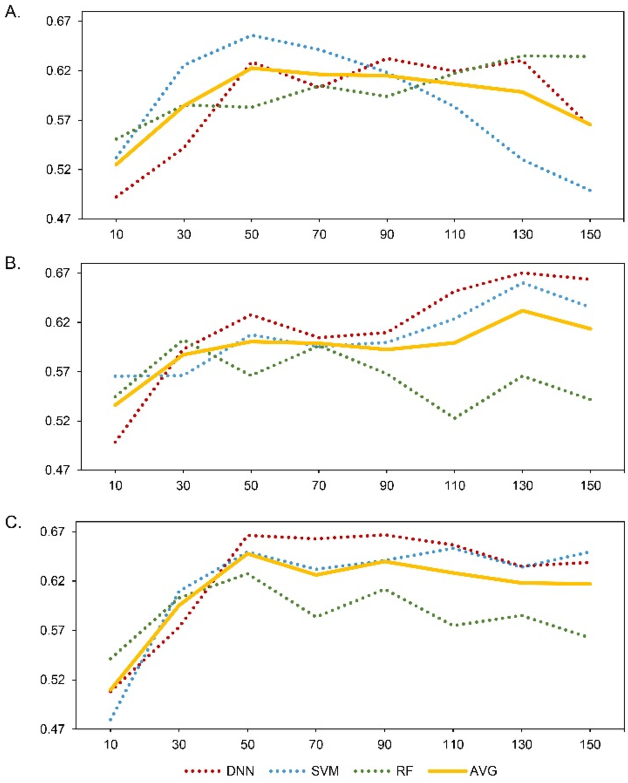

Figure S5: Classification performance of the feature selection method, joint deep semi-NMF model (JDSNMF), and other dimension reduction models using three classifiers for the ADNI cohort. (A) NMF model. (B) Deep semi-NMF model. (C) JDSNMF model. Deep neural network (DNN), support vector machine (SVM),and random forest (RF) were used and area under the curve (AUC) between true positives and false positive rates were measured for classification performance

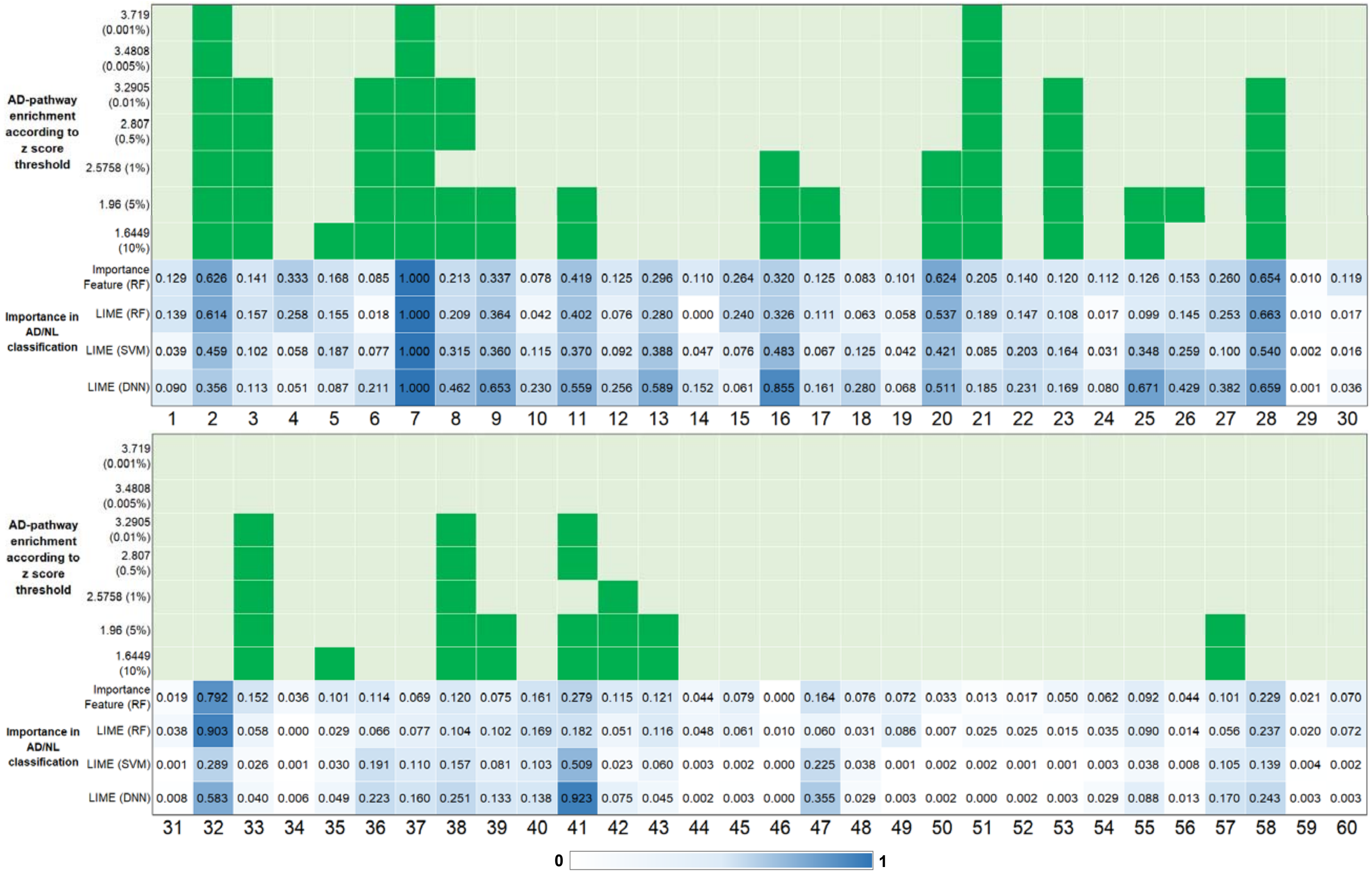

Figure S6: Identification of AD-related modules using the best performing three-layer model in the ANM validation set. The LIME model was used to score the importance of modules when DNN, SVM, and RF were used as classifiers. Using the Scikit-learn, important modules were scored when RF was used as a classifier. To validate the relationship between AD-related modules and AD, we constructed modules by including genes according to various z-score thresholds and performed enrichment analysis. Green elements indicate modules including the significantly enriched AD KEGG pathway.

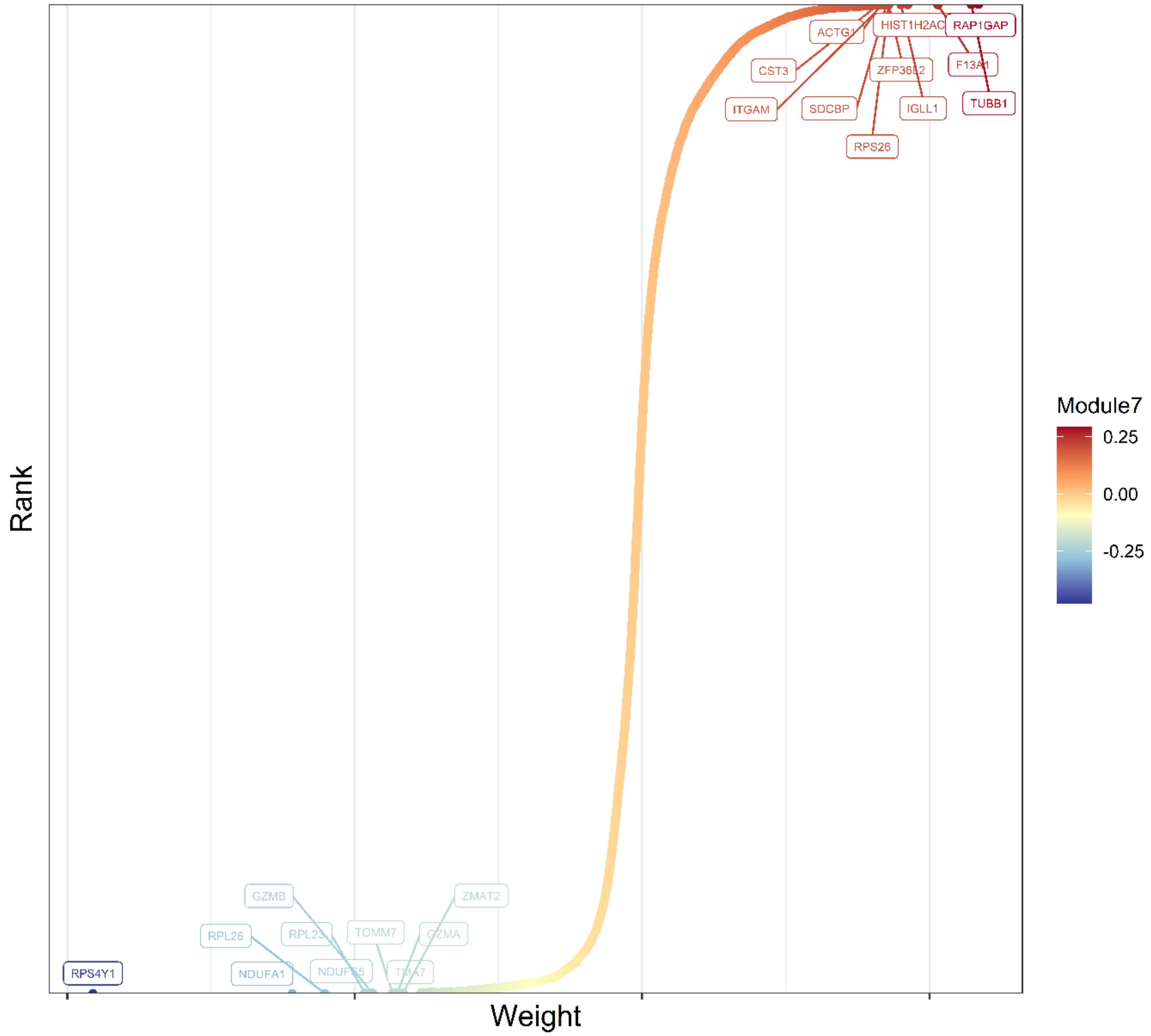

Figure S7: Top 10 of positive genes and negative genes in the AD-related module (module 7 of the best performing model in the ANM validation set).

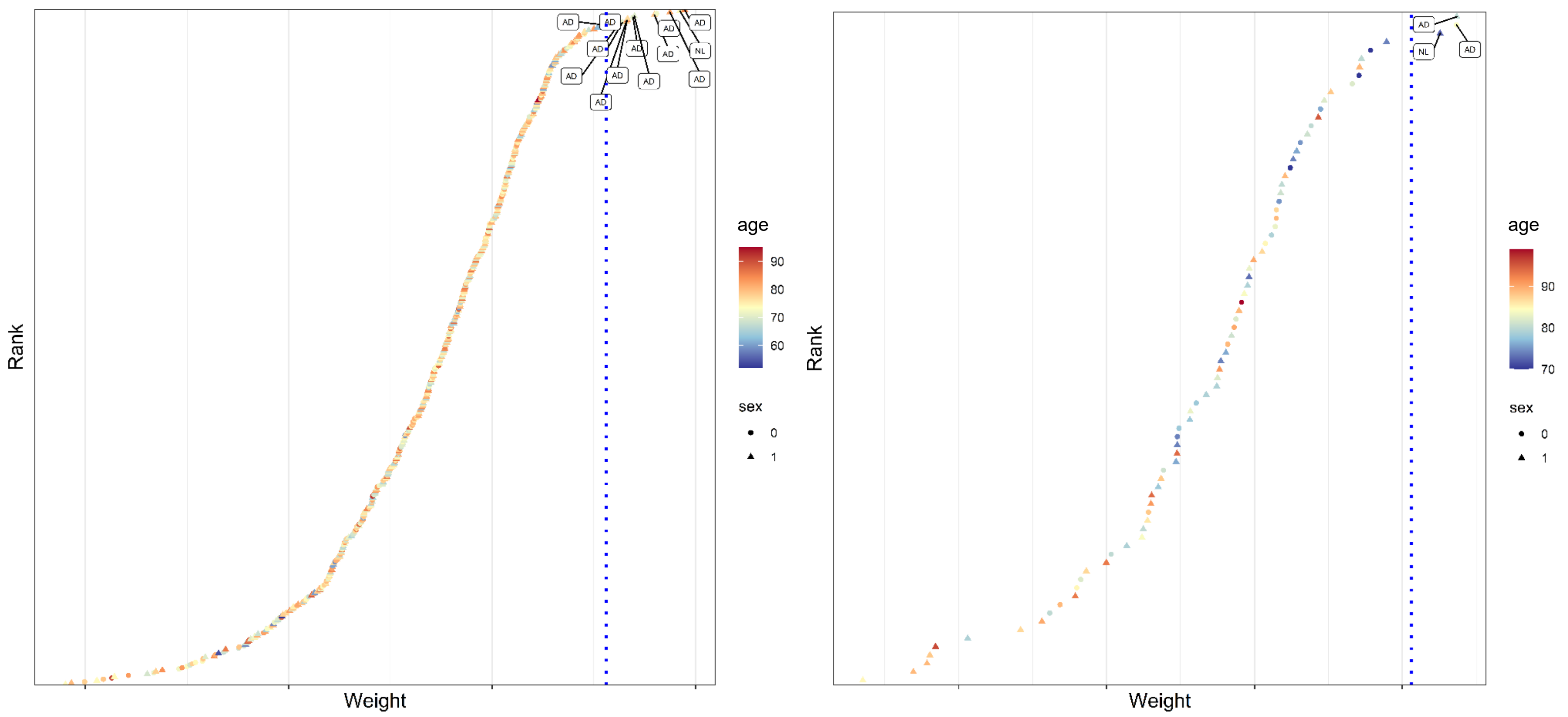

Figure S8: Samples larger than a predefined positive threshold (empirically set to μ + 1.6449σ, 5%) in the AD-related module (module 7 of the best performing model in the ANM validation set). The module 7 contains 12 AD samples and 1 NL sample in Gene expression data and 2 AD samples (left) and 1 NL sample in DNA methylation data

(right).

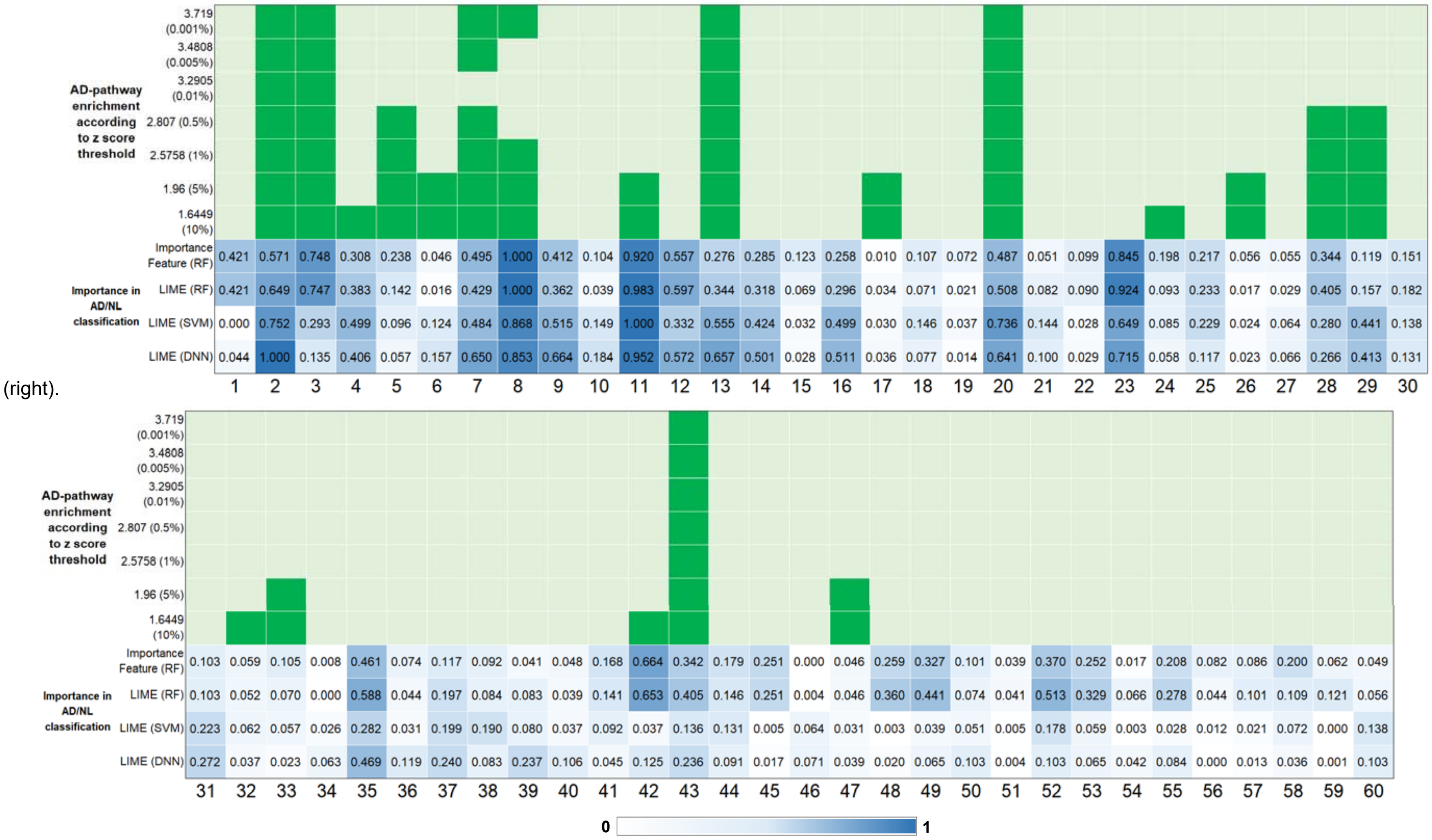

Figure S9: Identification for AD-related module using best performing two-layer model in the ANM validation set. The LIME model was used to score the importance of modules when DNN, SVM, and RF were used as classifiers. Using the Scikit-learn, important modules were scored when RF was used as a classifier. To validate the relationship between AD-related modules and AD, we constructed modules by including genes according to various z-score thresholds and performed enrichment analysis. Green elements indicate modules including the significantly enriched AD KEGG pathway.

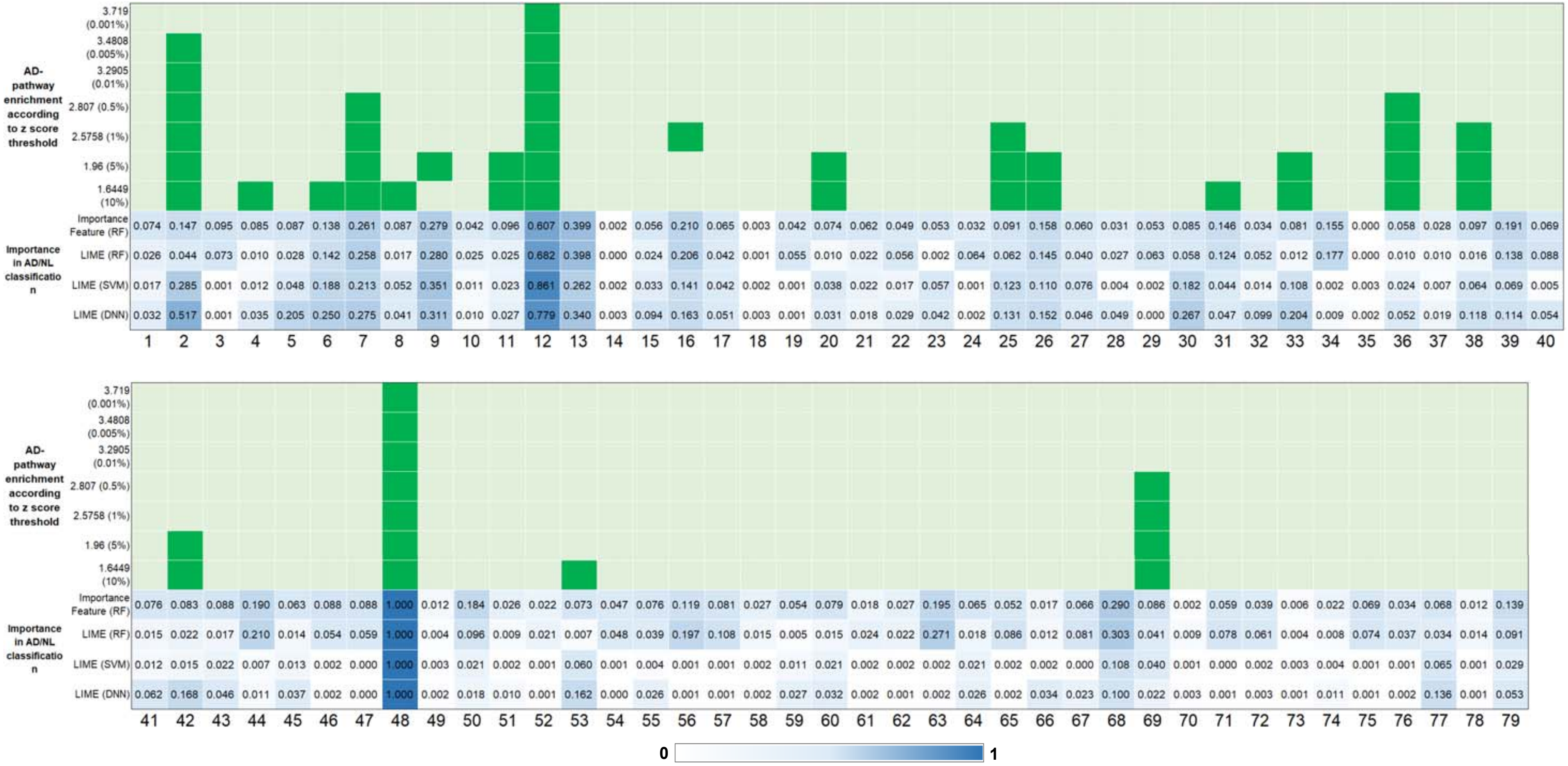

Figure S10: Identification for AD-related modules using the best performing four-layer model in the ANM validation set. The LIME model was used to score the importance of modules when DNN, SVM, and RF were used as classifiers. Using the Scikit-learn, important modules were scored when RF was used as a classifier. To validate the relationship between AD-related modules and AD, we constructed modules by including genes according to various z-score thresholds and performed enrichment analysis. Green elements indicate modules including the significantly enriched AD KEGG pathway.

	1	2	3	4	5	6	7	8	9	10	11	12	13	14	15
Age Coefficient (γ) GE	6.73E-04	1.36E-03	-6.20E-05	9.61E-05	6.82E-04	-2.55E-04	2.28E-04	-5.91E-04	-2.11E-04	-5.78E-04	-4.47E-04	-6.36E-04	5.22E-04	-1.46E-04	4.26E-04
Age Coefficient (γ) ME	-1.72E-03	1.58E-04	3.00E-04	1.84E-03	-8.04E-04	2.27E-04	-2.54E-04	-3.78E-04	3.66E-04	-7.94E-04	2.75E-04	1.26E-04	-4.30E-05	-2.82E-04	-2.96E-04
P-value for age in the regression GE	4.58E-03	1.18E-01	8.08E-01	6.26E-01	1.25E-02	2.38E-01	3.93E-01	1.67E-01	6.31E-01	3.64E-02	7.93E-02	4.36E-02	8.89E-02	5.47E-01	6.78E-02
P-value for age in the regression ME	3.53E-02	8.33E-02	4.17E-01	2.34E-01	2.78E-02	7.04E-02	3.58E-02	1.42E-01	3.43E-01	2.77E-01	2.07E-01	3.61E-01	7.42E-01	4.55E-01	2.99E-01

	16	17	18	19	20	21	22	23	24	25	26	27	28	29	30
Age Coefficient (γ) GE	-1.57E-03	-3.62E-04	-3.99E-04	1.97E-04	3.86E-04	-2.40E-04	6.50E-04	5.07E-04	-3.45E-04	5.46E-04	4.29E-04	-5.40E-05	-2.09E-03	5.53E-06	-4.90E-04
Age Coefficient (γ) ME	3.29E-04	6.38E-05	1.65E-03	-3.44E-04	2.90E-05	2.00E-04	-1.73E-03	-2.28E-05	-1.25E-03	-4.89E-06	-1.64E-04	2.89E-04	1.64E-04	2.91E-03	1.37E-04
P-value for age in the regression GE	2.07E-03	3.22E-01	1.20E-01	5.66E-01	5.10E-01	4.42E-01	2.57E-02	2.01E-01	2.73E-01	2.19E-01	2.10E-01	7.58E-01	1.15E-03	5.82E-01	8.71E-02
P-value for age in the regression ME	4.45E-01	8.29E-01	2.66E-02	5.04E-01	8.88E-01	4.20E-01	2.18E-02	8.99E-01	4.16E-02	9.77E-01	4.59E-01	7.93E-01	3.08E-01	4.96E-02	7.68E-01

	31	32	33	34	35	36	37	38	39	40	41	42	43	44	45
Age Coefficient (γ) GE	6.42E-06	-2.02E-04	-4.95E-04	1.53E-05	-9.61E-05	-4.41E-04	1.21E-03	-1.79E-04	2.56E-04	4.42E-04	4.93E-04	-6.38E-04	-6.98E-04	2.38E-06	-2.97E-05
Age Coefficient (γ) ME	2.47E-03	1.02E-04	-2.40E-04	2.61E-03	-1.16E-03	-8.86E-05	9.61E-04	4.45E-04	8.26E-04	-3.80E-05	-7.55E-04	6.46E-05	2.59E-05	-3.38E-03	4.65E-03
P-value for age in the regression GE	2.89E-01	5.09E-01	1.42E-01	1.06E-01	7.35E-01	3.94E-01	6.75E-05	5.68E-01	2.91E-01	6.60E-02	1.35E-01	4.35E-02	2.14E-02	6.41E-01	1.31E-01
P-value for age in the regression ME	1.19E-01	8.84E-01	8.03E-02	5.67E-02	1.40E-02	7.58E-01	2.36E-01	1.48E-01	3.47E-01	9.65E-01	1.54E-01	3.02E-01	9.14E-01	9.84E-02	1.59E-02

	46	47	48	49	50	51	52	53	54	55	56	57	58	59	60
Age Coefficient (γ) GE	3.17E-06	6.56E-04	-1.98E-04	-9.21E-06	9.13E-06	4.16E-06	1.38E-07	-3.08E-06	1.10E-04	-1.11E-04	-2.41E-04	6.84E-04	-7.51E-05	5.96E-07	1.40E-05
Age Coefficient (γ) ME	-3.44E-03	3.77E-04	-1.79E-03	-4.82E-03	-2.86E-05	-5.04E-04	-3.58E-03	2.26E-03	3.21E-03	4.44E-04	-1.05E-03	5.18E-05	2.91E-04	4.39E-03	-1.47E-03
P-value for age in the regression GE	5.31E-01	2.18E-01	3.07E-01	2.39E-01	1.11E-01	4.87E-01	9.73E-01	9.02E-01	3.74E-01	6.28E-01	1.34E-01	7.32E-02	8.41E-01	8.98E-01	2.58E-01
P-value for age in the regression ME	7.61E-02	5.33E-01	1.45E-01	2.68E-04	9.88E-01	7.76E-01	5.91E-02	9.58E-02	5.65E-02	7.49E-01	3.91E-01	3.88E-01	3.47E-01	1.87E-03	3.90E-01

Figure S11: Identification of modules for the relation to age. The linear regression was used to identify statistically significant modules related to age. Green elements indicate modules with p-value < 0.05.

Term	Overlap	P.value	Adjusted.P. value
neutrophil mediated immunity (GO:0002446)	35/487	1.48E-17	2.51E-14
neutrophil activation involved in immune response (GO:0002283)	35/483	1.14E-17	2.91E-14
neutrophil degranulation (GO:0043312)	35/479	8.79E-18	4.49E-14
secretory granule lumen (GO:0034774)	28/317	1.68E-16	7.50E-14
specific granule (GO:0042581)	16/160	9.82E-11	2.19E-08
specific granule lumen (GO:0035580)	11/62	1.75E-10	2.60E-08
cytoplasmic vesicle lumen (GO:0060205)	13/129	5.31E-09	5.92E-07
Antigen processing and presentation	10/77	2.79E-08	2.86E-06
Type I diabetes mellitus	8/43	3.86E-08	2.97E-06
Graft-versus-host disease	8/41	2.60E-08	4.00E-06
MHC protein complex (GO:0042611)	6/18	4.62E-08	4.12E-06
Allograft rejection	8/38	1.37E-08	4.22E-06
Autoimmune thyroid disease	8/53	2.13E-07	1.31E-05
Hematopoietic cell lineage	10/97	2.59E-07	1.33E-05
MHC class II protein complex (GO:0042613)	5/14	4.37E-07	3.25E-05
Influenza A	12/171	1.11E-06	3.79E-05
defense response to bacterium (GO:0042742)	16/241	3.81E-08	3.89E-05
defense response to fungus (GO:0050832)	6/17	3.11E-08	3.96E-05
Toxoplasmosis	10/113	1.08E-06	4.16E-05
Epstein-Barr virus infection	13/201	9.95E-07	4.38E-05
tertiary granule (GO:0070820)	12/164	7.10E-07	4.52E-05
Staphylococcus aureus infection	8/68	1.52E-06	4.68E-05
Leishmaniasis	8/74	2.92E-06	8.16E-05
Viral myocarditis	7/59	6.59E-06	0.00016902
tertiary granule lumen (GO:1904724)	7/55	4.08E-06	0.00022745
Inflammatory bowel disease (IBD)	7/65	1.26E-05	0.00029952
Phagosome	10/152	1.55E-05	0.00034184
Systemic lupus erythematosus	9/133	3.32E-05	0.00068249
azurophil granule (GO:0042582)	10/154	1.74E-05	0.00086287
defense response to Gram-positive bacterium (GO:0050830)	8/65	1.07E-06	0.00091102
integral component of lumenal side of endoplasmic reticulum membrane (GO:0071556)	5/29	2.24E-05	0.00099903
vacuolar lumen (GO:0005775)	10/161	2.56E-05	0.00103726
Tuberculosis	10/179	6.30E-05	0.00121301
defense response to Gram-negative bacterium (GO:0050829)	8/71	2.12E-06	0.00135382
interferon-gamma-mediated signaling pathway (GO:0060333)	8/70	1.90E-06	0.00138711
endocytic vesicle lumen (GO:0071682)	4/18	5.42E-05	0.0020149
Th1 and Th2 cell differentiation	7/92	0.00012094	0.00219117
ficolin-1-rich granule (GO:0101002)	10/184	7.93E-05	0.0027223
azurophil granule lumen (GO:0035578)	7/90	0.00010526	0.00335315
ficolin-1-rich granule lumen (GO:1904813)	8/123	0.00012039	0.00357952
B cell receptor signaling pathway	6/71	0.00020944	0.00358368
MHC class II receptor activity (GO:0032395)	4/10	4.01E-06	0.00461858
Th17 cell differentiation	7/107	0.00030928	0.00501356
Human T-cell leukemia virus 1 infection	10/219	0.00032757	0.00504456
Cell adhesion molecules (CAMs)	8/145	0.00037161	0.00520247
primary lysosome (GO:0005766)	3/10	0.00019241	0.00536345
PI3K-Akt signaling pathway	13/354	0.0003699	0.00542515
Kaposi sarcoma-associated herpesvirus infection	9/186	0.0004261	0.00570608
Legionellosis	5/55	0.00050923	0.00627373
Asthma	4/31	0.00049323	0.00632973
Pathways in cancer	16/530	0.00070495	0.00835098
Rheumatoid arthritis	6/91	0.00079898	0.00911424
IL-17 signaling pathway	6/93	0.00089572	0.00985288
Primary immunodeficiency	4/37	0.00097861	0.01039352
Human immunodeficiency virus 1 infection	9/212	0.00108471	0.01077707
Amoebiasis	6/96	0.00105745	0.01085651
Acute myeloid leukemia	5/66	0.00117579	0.01131699
ER to Golgi transport vesicle membrane (GO:0012507)	5/54	0.00046746	0.01226391
antimicrobial humoral immune response mediated by antimicrobial peptide (GO:0061844)	6/49	2.55E-05	0.01299853
Longevity regulating pathway	6/102	0.00144734	31
Adipocytokine signaling pathway	5/69	0.00143627	0.01340515
positive regulation of NF-kappaB transcription factor activity (GO:0051092)	9/128	2.45E-05	0.01390407
Human cytomegalovirus infection	9/225	0.00163802	0.01441453
Transcriptional misregulation in cancer	8/186	0.00187413	0.01603421
Chemokine signaling pathway	8/190	0.00214035	0.01781693
C3HC4-type RING finger domain binding (GO:0055131)	3/6	3.32E-05	0.01912441
lysosomal membrane (GO:0005765)	11/291	0.00081898	0.02029239
Intestinal immune network for IgA production	4/48	0.00260133	0.02108443
Viral carcinogenesis	8/201	0.00302775	0.02391144
Protein processing in endoplasmic reticulum	7/165	0.00384253	0.02958748
Osteoclast differentiation	6/127	0.00434125	0.03261229
Glutathione metabolism	4/56	0.00455763	0.03342263
Natural killer cell mediated cytotoxicity	6/131	0.00504593	0.03614295
cellular response to interferon-gamma (GO:0071346)	8/116	7.98E-05	0.03701505
tertiary granule membrane (GO:0070821)	5/73	0.00184672	0.04118189
lysosome (GO:0005764)	13/422	0.00184451	0.04329746
COPII-coated ER to Golgi transport vesicle (GO:0030134)	5/75	0.00208139	0.04420466
Measles	6/138	0.00647607	0.0453325

Table S2: Significantly enriched gene ontology terms and KEGG pathways of genes in the module 1.
